# Sensitive Detection of p65 Homodimers Using Red-Shifted and Fluorescent Protein-Based FRET Couples

**DOI:** 10.1371/journal.pone.0001011

**Published:** 2007-10-10

**Authors:** Joachim Goedhart, Joop E. M. Vermeer, Merel J. W. Adjobo-Hermans, Laura van Weeren, Theodorus W. J. Gadella

**Affiliations:** Section of Molecular Cytology, Centre for Advanced Microscopy, Swammerdam Institute for Life Sciences, University of Amsterdam, Amsterdam, The Netherlands; University of Washington, United States of America

## Abstract

**Background:**

Fluorescence Resonance Energy Transfer (FRET) between the green fluorescent protein (GFP) variants CFP and YFP is widely used for the detection of protein-protein interactions. Nowadays, several monomeric red-shifted fluorescent proteins are available that potentially improve the efficiency of FRET.

**Methodology/Principal Findings:**

To allow side-by-side comparison of several fluorescent protein combinations for detection of FRET, yellow or orange fluorescent proteins were directly fused to red fluorescent proteins. FRET from yellow fluorescent proteins to red fluorescent proteins was detected by both FLIM and donor dequenching upon acceptor photobleaching, showing that mCherry and mStrawberry were more efficient acceptors than mRFP1. Circular permutated yellow fluorescent protein variants revealed that in the tandem constructs the orientation of the transition dipole moment influences the FRET efficiency. In addition, it was demonstrated that the orange fluorescent proteins mKO and mOrange are both suitable as donor for FRET studies. The most favorable orange-red FRET pair was mKO-mCherry, which was used to detect homodimerization of the NF-κB subunit p65 in single living cells, with a threefold higher lifetime contrast and a twofold higher FRET efficiency than for CFP-YFP.

**Conclusions/Significance:**

The observed high FRET efficiency of red-shifted couples is in accordance with increased Förster radii of up to 64 Å, being significantly higher than the Förster radius of the commonly used CFP-YFP pair. Thus, red-shifted FRET pairs are preferable for detecting protein-protein interactions by donor-based FRET methods in single living cells.

## Introduction

Fluorescent protein fusions are widely used to study the localization and dynamics of proteins in living cells [1], [2]. The development of spectral variants allows the study of multiple fluorescent protein fusions at the same time in a single cell [3], [4]. Moreover, spectral variants can be used to monitor protein-protein interactions or conformational changes by means of Fluorescence Resonance Energy Transfer (FRET) [5], [6]. FRET is the process in which an excited (donor) fluorophore relaxes back to the ground state by transferring its energy radiationless to another (acceptor) chromo- or fluorophore [7], [8]. The most popular fluorescent protein pair for measuring interactions or conformation changes consists of Cyan Fluorescent Protein (CFP) as the donor and Yellow Fluorescent Protein (YFP) as the acceptor. Several improvements in the spectral properties of CFP and YFP have been made [9]–[12] which have increased the FRET efficiency of this couple.

The application of the CFP/YFP couple for detecting FRET has been very successful, yet some characteristics of this couple are not optimal. First, the blue excitation necessary for CFP can induce considerable levels of autofluorescence. Second, the multi-exponential decay of CFP complicates the analysis of FRET by lifetime measurements. In addition, the fluorescent proteins may undergo photoconversion or reversible photobleaching [13]. By moving the excitation wavelength towards the red, autofluorescence levels generally decrease.

Another advantage of red-shifted couples is the fact that the FRET efficiency generally increases for pairs at higher wavelengths. This is caused by a larger Förster radius due to a λ^4^ dependence in the overlap integral J(λ) of the Förster equation (R_0_ in Å):
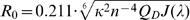
(1)in which κ^2^ is the orientation factor, n is the refractive index of the medium, Q_D_ is the quantum yield of the donor and J(λ) (in M^−1^ cm^−1^ nm^4^) is defined as:
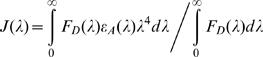
(2)F_D_(λ) is the fluorescence emission spectrum of the donor, ε_A_(λ) is the absorbance spectrum of the acceptor and λ is the wavelength [8].

A first step towards red-shifted FRET couples was the identification of a red fluorescent protein, DsRed [14]. However, the existence of a green intermediate state in the maturation and tetramerization of the red fluorescent protein was a serious problem for FRET applications. The development of monomeric Red Fluorescent Protein (mRFP1) solved the problems of slow and incomplete maturation and obligate tetramerization of DsRed [15]. Subsequently, mRFP1 has been improved to yield novel red fluorescent proteins, named mCherry and mStrawberry, with increased photostability, maturation rate and extinction coefficient [16]. Due to their relatively high extinction coefficient these proteins are attractive FRET acceptors for yellow/orange donors. Although some studies have appeared that use yellow and red fluorescent proteins for FRET studies [17]–[20] a detailed side-by-side comparison of several combinations for the detection of FRET in single living cells is still lacking. Therefore, our aim was to explore whether red-shifted FRET couples provide superior alternatives to the CFP/YFP couple for the detection of protein-protein interactions in single living cells.

To this end, a series of tandem constructs were made in which a donor was fused directly to an acceptor, while keeping the linker equal to allow an as fair as possible comparison between pairs. These tandem constructs allow straightforward comparison of FRET efficiencies between different pairs since, (i) the FRET pair is present in a 1∶1 expression, and (ii) the distance/orientation between the constructs is as similar as possible due to equal linkers. Similar approaches have been taken to characterize FRET in CFP-YFP pairs and these tandem constructs can be potentially useful as FRET standards [21], [22].

A very robust way of measuring FRET in living cells is the determination of the excited state lifetime of the donor fluorophore by fluorescence lifetime imaging microscopy (FLIM) [23]–[25]. Therefore FLIM was used to quantify the FRET efficiencies of the pairs. In addition, circularly permutated YFP variants were used as donors to study possible effects of orientation on the FRET efficiencies. Finally the FRET pair that was found to be the most efficient, based on R_0_ and on FRET measurements of tandem constructs in cells, was used to measure the homodimerization of the NF-κB subunit p65 in living cells.

## Results

### Calculation of the Förster radii

The Förster radius (R_0_) of a FRET pair is defined as the distance at which 50% of the energy transfer takes place and it is the principal quality measure for a FRET pair. Since we consistently observed higher fluorescence emission for purified mKO relative to mOrange at equal absorbance, we re-evaluated their quantum yields. For mOrange we found a value of 0.67 which is equal to the published value [16]. However, for mKO we found a value of 0.74 which is substantially higher than the published value of 0.60 [26]. We currently do not have an explanation for this discrepancy and the value of 0.74 was used for the calculation of the Förster radii. As for the donor quantum yield and acceptor extinction coefficient of the other fluorescent proteins their published values were used [10], [16].

The Förster radius R_0_ and overlap integral J(λ) of the FRET pairs used in this study were calculated according to equation 1 and 2 respectively and are shown in table 1. The refractive index of water was used (n = 1.33) and we set the orientation factor κ^2^ to 2/3 (representing random donor and acceptor dipole moment orientations) to allow comparison across the literature. It is of note that the calculated R_0_ values may differ from actual R_0_ values due to a different value of the orientation factor [24]. Still, the presented R_0_ values allow an unbiased quantitative comparison to the Förster radii that have been calculated for other FRET pairs. The overlap integral can be used to calculate the R_0 _for other values of κ^2^ and n using equation 1.

**Table 1:** Overlap integral and Förster radius of the fluorescent protein pairs used in this study.

Donor	Acceptor	J(λ)*10^−15^ M^−1^ cm^−1^ nm^4^	R_0_ [Å]
SYFP2	mRFP1	2.29	56
SYFP2	mStrawberry	4.90	63
SYFP2	mCherry	3.14	59
mOrange	mRFP1	3.28	59
mOrange	mCherry	4.81	63
mKO	mRFP1	3.26	60
mKO	mCherry	4.79	64

It can be inferred from the table that all pairs have an R_0_ value that is substantially higher than the R_0_ of the popular ECFP-EYFP pair (around 47 Å), and higher than that of the improved SCFP3A-SYFP2 pair (54 Å) [10]. This can be explained by the higher quantum yield of the yellow and orange donors as compared to ECFP and SCFP3A and by the λ^4^ component in the overlap integral which generally increases the Förster radius for pairs in the red part of the visible spectrum. Based on the R_0_ values, mStrawberry is the preferred acceptor for SYFP2 and the orange-red pair with the highest R_0_ of 64 Å is mKO-mCherry.

### Construction of the fluorescent protein pairs

**Figure 1:**
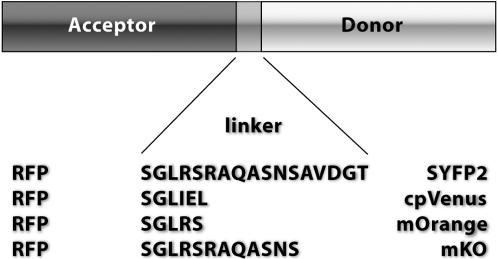
Schematic overview of the tandem fluorescent protein constructs used in this study. The red fluorescent protein, abbreviated as RFP, is either mRFP1, mStrawberry or mCherry. Single letter abbreviations are used for the amino acids that comprise the linker.

Tandem fusion proteins with the red fluorescent proteins, mRFP1 [15], mStrawberry and mCherry [16] as FRET acceptors, were constructed as indicated in figure 1. As donors we used the recently described yellow fluorescent protein variant SYFP2 [10] or the orange fluorescent proteins mKO [26] and mOrange [16]. Similar linkers within a group of pairs were used to avoid linker dependent differences in FRET efficiency between pairs. Details of the fluorescent protein pairs and the sequence of the linkers can be found in the supplementary table (Table S1).

### FLIM of YFP donor fusion proteins

The tandem fluorescent protein fusions carrying a red fluorescent protein as acceptor were expressed in HeLa cells. One day after transfection, the FRET efficiency in living cells was examined using frequency domain Fluorescence Lifetime Imaging Microscopy (FLIM). This approach yields two lifetimes, a phase lifetime (τ_ϕ_) determined from the phase shift of the emission light and a modulation lifetime (τ_Μ_) determined from decrease in modulation depth of the emission light relative to the excitation light [23]. The FRET efficiency of a tandem construct can be calculated from the decreased donor lifetime (τ_ϕ_ or τ_Μ_) relative to the fluorescence lifetime of an unquenched donor according to equation 3. This yields two FRET efficiency values, one based on τ_ϕ_ and another based on τ_Μ_.

**Figure 2:**
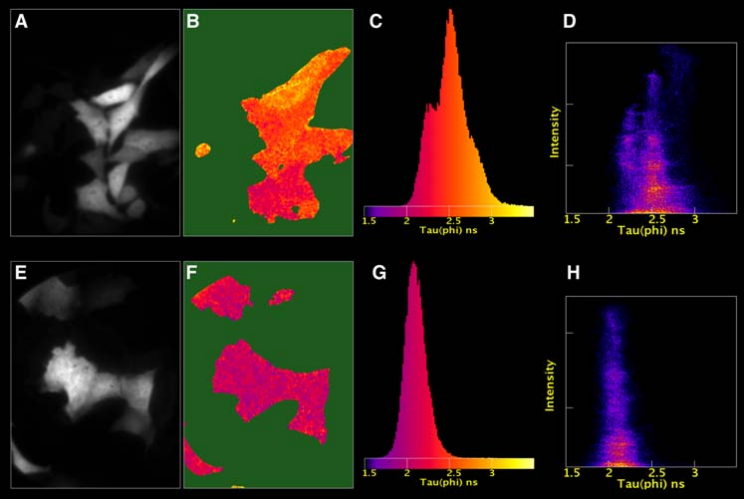
FLIM data of living cells expressing the mStrawberry-SYFP2 tandem. Experiments were performed either 1 day (a–d) or 2 days (e–h) after transfection. The panels show the fluorescence intensity of SYFP2 (a, e), the phase lifetime map of SYFP2 (b, f), the histogram of the lifetime distribution (c, g) and the 2D histogram of the lifetime distribution versus the fluorescence intensity (d, h). The false color representation of the lifetime map corresponds to the colors used in the lifetime histogram. The width of the images corresponds to 112 µm.

Cells that expressed only the donor (SYFP2) showed a homogeneous phase lifetime around 3.1 ns similar to the value described before [10]. However, cells expressing the mStrawberry-SYFP2 tandem showed a decreased phase lifetime that varied significantly from 2.2 ns up to 2.8 ns (figure 2a–d). From the lifetime map (figure 2b) it is clear that the variation is caused by differences between individual cells rather than differences within a single cell. There is no correlation between fluorescence intensity of cells and their fluorescence lifetime as can be inferred from the 2D histogram (figure 2d) in which the intensity is plotted against the lifetime on a pixel-by-pixel basis. The variation in lifetime indicates a variation in the FRET efficiency probably caused by incomplete protein folding/maturation of the acceptor. Since it is reported that the maturation of mStrawberry is relatively slow, FLIM experiments were performed two and three days after transfection. Two days after transfection cells expressing mStrawberry-SYFP2 displayed low and homogeneously distributed phase lifetime of 2.1 ns (figure 2e–h) which again was independent of fluorescence intensity (figure 2h). The same result was obtained three days after transfection (data not shown). The variation was specific for the mStrawberry acceptor as the mRFP1 and mCherry tandems showed little cell to cell variation one day after transfection. Interestingly, we did not find heterogeneity in the fluorescence lifetime when the YFP-mStrawberry pair was used to study the interaction of heterotrimeric G-proteins in plant cells [17]. These cells are incubated overnight at room temperature after transfection, suggesting that in this system the lower temperature is beneficial for the folding or maturation of the mStrawberry.

The results of the FLIM analysis of the fusion proteins with SYFP2 as the donor and the red fluorescent proteins as acceptor are shown in table 2. Both phase and modulation lifetime are clearly reduced in the tandem fusion constructs, indicating FRET. The donor lifetime is reduced to a greater extent in the mCherry and mStrawberry constructs relative to the mRFP1 construct. The higher FRET efficiency (calculated according to equation 3) for mCherry and mStrawberry acceptors is most likely due to the higher extinction coefficient (ε_A_, also see equation 2) of the next generation of red fluorescent proteins. Therefore, the mCherry and mStrawberry are more efficient than mRFP1 as FRET acceptors.

**Table 2:** Fluorescence Lifetime Imaging data from the FRET pairs containing SYFP2 as the donor fluorescent protein.

Donor	Acceptor	n^1^	τ_ϕ_ [ns]^2^	τ_M_ [ns]^3^	E_τϕ_ [%]^4^	E_τM_ [%]^4^
SYFP2	-	33	3.09±0.05	3.20±0.05	-	-
SYFP2	mRFP1	25	2.36±0.04	2.74±0.06	24	14
SYFP2	mStrawberry	26	2.12±0.11	2.56±0.09	31	20
SYFP2	mCherry	29	2.14±0.05	2.58±0.09	31	19

1 n number of cells from which the lifetime is calculated

2 τ_ϕ_ average phase lifetime±standard deviation

3 τ_M_ average modulation lifetime±standard deviation

4 E average FRET efficiency calculated from τ_ϕ_ or τ_M_

### Acceptor photobleaching of red acceptors

The same constructs were examined for FRET by acceptor photobleaching [27] using the 568 nm line of a Ar/Kr laser on a commercial laser scanning microscope. For all three pairs, a clear increase in yellow fluorescence was observed after bleaching of the acceptor. The FRET efficiency was calculated using equation 4 and is listed in table 3. When compared to the apparent FRET values calculated from the FLIM experiments (table 2), it is clear that the overall trend is the same, with mCherry and mStrawberry being substantially better FRET acceptors than mRFP1. The FRET efficiency calculated from the acceptor bleaching experiments is significantly higher than that calculated from lifetime values. This is a known phenomenon, which can be explained by the fact that the contributing lifetimes are not equally weighed in the determined lifetimes. As a result, the average lifetime determined by FLIM is biased towards the higher lifetimes of multi-exponentially decaying donors. A comprehensive discussion can be found elsewhere [28], [29]. In conclusion, acceptor bleaching is very well suited to identify FRET from SYFP2 to the red fluorescent acceptors.

**Table 3:** FRET efficiency of FRET pairs containing SYFP2 as the donor fluorescent protein calculated from donor dequenching by acceptor photobleaching.

Donor	Acceptor	n^1^	E [%]
SYFP2	-		-
SYFP2	mRFP1	17	32±1
SYFP2	mStrawberry	22	41±2
SYFP2	mCherry	24	38±2

1 n number of cells analyzed

2 E average FRET efficiency±standard deviation

### FLIM of circularly permutated YFP donor fusion proteins

Since FRET is an orientation dependent process, we investigated the role of the relative chromophore orientation in the tandem constructs. To this end, we took advantage of the availability of circularly permutated variants of the YFP variant Venus. These variants have new N- and C-termini introduced at five different places in the beta-barrel, thereby changing the orientation of the donor fluorophore [30].

First of all, the fluorescence lifetime of the donors only, including non-permutated Venus, were measured. The results are summarized in table 4. Interestingly, both phase and modulation lifetimes of the circular permutated proteins were higher compared to Venus, except for cpV6 which has a fluorescence lifetime similar to Venus of 2.9 ns. The highest lifetime was observed for cpV9 of which the phase and modulation lifetime is increased by 0.23 ns and 0.28 ns respectively.

**Table 4:** FLIM data from the FRET pairs containing circularly permutated yellow fluorescent protein as the donor.

Donor	Acceptor	n^1^	τ_ϕ_ [ns]^2^	τ_M_ [ns]^3^	E_τϕ_ [%]^4^	E_τM_ [%]^4^
Venus	-	8	2.91±0.05	2.94±0.05	-	-
cpV2	-	23	2.98±0.06	3.19±0.07	-	-
cpV3	-	21	3.04±0.07	3.20±0.06	-	-
cpV6	-	28	2.87±0.04	2.92±0.08	-	-
cpV7	-	23	3.00±0.07	3.15±0.05	-	-
cpV9	-	23	3.14±0.06	3.22±0.04	-	-
cpV2	mCherry	23	2.00±0.04	2.42±0.04	33	24
cpV3	mCherry	25	1.77±0.09	2.27±0.06	42	29
cpV6	mCherry	21	1.70±0.03	2.23±0.04	41	24
cpV7	mCherry	25	1.85±0.06	2.36±0.06	38	25
cpV9	mCherry	22	2.20±0.07	2.60±0.08	30	19

1 n number of cells from which the lifetime is calculated

2 τ_ϕ_ average phase lifetime±standard deviation

3 τ_M_ average modulation lifetime±standard deviation

4 E average FRET efficiency calculated from τ_ϕ_ or τ_M_

FLIM of the mCherry-cpV constructs shows a clearly reduced phase and modulation lifetime for all constructs (table 4). In general, the FRET efficiency between the Cherry-cpV constructs is higher than that of the mCherry-SYFP2 construct, probably due to the longer linker in the latter construct. Phase and modulation lifetimes decreased most significantly in the mCherry-cpV3 construct, yielding FRET efficiencies of 42% and 29% calculated based on phase and modulation lifetimes respectively. The lowest FRET efficiencies were calculated for mCherry-cpV9, 30% and 19% calculated based on phase and modulation lifetimes respectively. Thus, the efficiency of FRET to the red fluorescent protein acceptor depends on permutation of the donor fluorophore, likely reporting on differences in orientation between donor and acceptor transition dipoles. These results suggest that it is worthwhile to explore the use of circularly permutated donor fluorophores in FRET studies on protein-protein interactions or in FRET-based reporters based on YFP and mCherry.

### FLIM of orange fluorescent proteins and photoconversion

**Figure 3:**
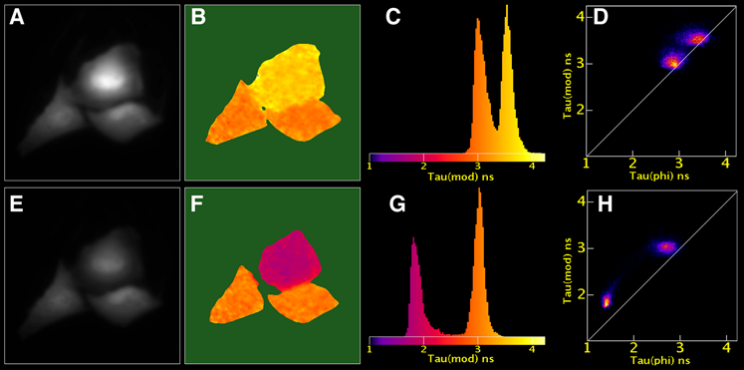
FLIM data of cells expressing mKO or mOrange. The FLIM was done before (a–d) and after (e–h) exposing the cells to 436 nm light. The panels show the fluorescence intensity (a, e), the modulation lifetime map (b, f), the histogram of the modulation lifetime distribution (c, g) and the 2D histogram of the phase lifetime versus the modulation lifetime (d, h). The false color representation of the lifetime map corresponds to the colors used in the lifetime histogram. The width of the images corresponds to 85 µm.

Since the FRET efficiency is dependent on the overlap between donor emission and acceptor absorbance spectra, we took orange donors that are red-shifted relative to YFP. The efficiency of FRET to a red acceptor would be increased due to a larger overlap integral (see table 1). First, the putative orange fluorescent donors, mOrange and mKO, were characterized. The fluorescence lifetime of mOrange in living cells was τ_ϕ_ = 2.7 ns and τ_Μ_ = 2.9 ns. The fluorescence lifetime of mKO was τ_ϕ_ = 3.5 ns and τ_Μ_ = 3.7 ns which is a relatively high lifetime, as observed before [26]. A serendipitous discovery was made when mKO expressing cells were illuminated with intense 436 nm light from a mercury lamp. Surprisingly, these cells displayed green fluorescence rather than orange fluorescence. The green fluorescent species was stable for at least 30 minutes. When lifetime imaging was performed on cells after illumination with 436 nm light an average phase lifetime of 1.4±0.04 ns and modulation lifetime of 1.9±0.1 ns (n = 9) was obtained. To examine whether this is specific for mKO or a general feature of orange chromophores, a sample was prepared containing both mOrange and mKO expressing cells. A FLIM experiment on a mixed sample shows that a mKO expressing cell can be clearly discerned from mOrange expressing cells based on lifetime contrast (figure 3a–d). The presence of two different lifetime populations is also evident from the 1D histogram (figure 3c) and 2D histogram (figure 3d) in which the modulation lifetime is plotted against the phase lifetime on a pixel-by-pixel basis. After exposure to 436 nm light a striking lifetime contrast is observed between mOrange and mKO expressing cells excited at 514 nm (figure 3e–h). The mOrange expressing cells show a similar lifetime as non-exposed cells, but the lifetime of mKO expressing cells has dropped by almost 2 ns (compare figure 3f–h with figure 3b–d). In addition, the mKO fluorescence was reduced to 46% (n = 9), whereas mOrange was only reduced to 75% (n = 6) of the original intensity. Photoconversion of mKO to a green species is a first example in which, besides a color change, a lifetime contrast is observed. Interestingly, we also observed photoconversion of the original dimeric KO (data not shown), suggesting that the conversion is an intrinsic property of the mKO chromophore rather than a consequence of the extensive mutagenesis that was necessary to generate a monomeric protein [26]. Fortunately, no significant photoconversion is observed when mKO is exposed to light >500 nm. Therefore, this protein can be used reliably as a donor in FRET studies.

### FLIM of fusion proteins with orange fluorescent donors

Both mOrange and mKO were fused to mRFP1 and mCherry as acceptor fluorophores. The mStrawberry was not included as an acceptor since its emission spectrum largely overlaps with the emission spectrum of orange fluorescent proteins, leaving little spectral bandwith to specifically detect the orange donor. Results of the FLIM experiments on the tandem constructs are summarized in table 5. A clear reduction in lifetime is observed in all tandem constructs, relative to the donor-only measurements. Again, the tandem constructs containing mRFP1 as an acceptor show a smaller reduction than the mCherry constructs. The mCherry-mKO construct shows the largest reduction in donor fluorescence lifetime; 1.3 ns. The energy transfer in the constructs containing mOrange is lower than mKO based constructs. Thus, mKO is more efficient as a donor than mOrange for FRET to red fluorescent proteins.

**Table 5:** FLIM data from the FRET pairs containing orange fluorescent proteins as the donor.

Donor	Acceptor	n^1^	τ_ϕ_ [ns]^2^	τ_M_ [ns]^3^	E_τϕ_ [%]^4^	E_τM_ [%]^4^
mOrange	-	23	2.66±0.17	2.91±0.10	-	-
mOrange	mRFP1	27	2.07±0.04	2.55±0.05	22	12
mOrange	mCherry	20	1.88±0.06	2.34±0.07	29	20
mKO	-	20	3.46±0.06	3.73±0.06	-	-
mKO	mRFP1	23	2.47±0.04	3.09±0.05	29	17
mKO	mCherry	25	2.12±0.07	2.91±0.07	39	22

1 n number of cells from which the lifetime is calculated

2 τ_ϕ_ average phase lifetime±standard deviation

3 τ_M_ average modulation lifetime±standard deviation

4 E average FRET efficiency calculated from τ_ϕ_ or τ_M_

### Detection of protein-protein interaction using the mKO-mCherry FRET pair

**Figure 4:**
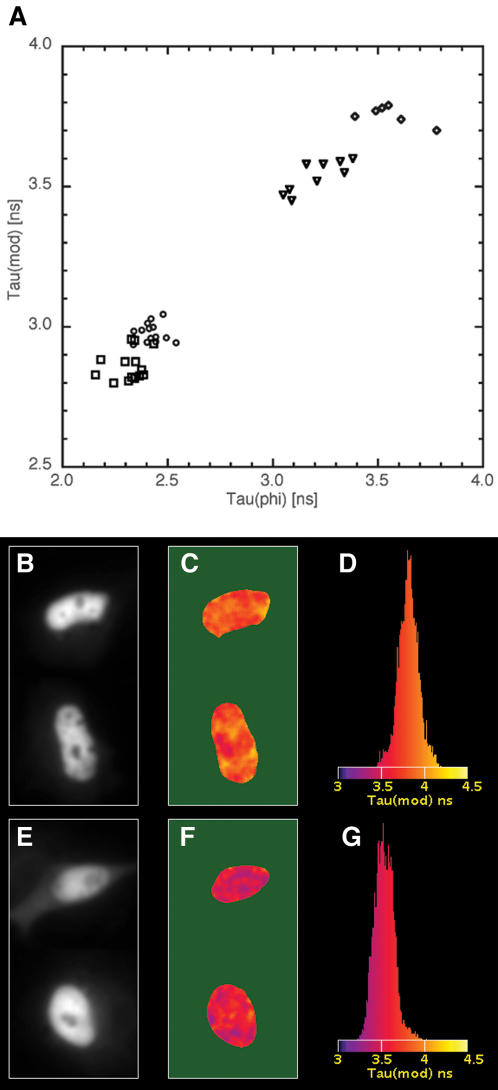
Homodimerization of p65 can be detected by FLIM. The lifetime data of multiple cells is summarized (a), by plotting the modulation lifetime against the phase lifetime for cells expressing ECFP-p65 (circles), ECFP-p65 and EYFP-p65 (squares), mKO-p65 only (diamonds) or cells expressing mKO-p65 and mCherry-p65 (triangles). FLIM images of cells expressing mKO-p65 in absence (b–d) or presence (e–g) of mCherry-p65. The panels show the fluorescence intensity of two merged representative nuclei (b, e), the modulation lifetime map (c, f) and the histogram of the modulation lifetime distribution (d, g). The reduced lifetime observed for cells expressing both mKO-p65 and p65-mCherry is due to FRET, indicating homodimerization of p65. The width of the images corresponds to 28 µm.

A previous study has shown that transcription factor homodimers can be detected by FLIM in single living cells [31]. Since it is known from structural studies and from biochemical experiments on cell extracts that the NF-κB transcription factor complex may exist as a p65 homodimer [32], our aim was to examine whether p65 homodimers can be detected in single living cells by FLIM, and to compare the suitability of CFP/YFP versus mKO-/mCherry. To this end, p65 was tagged with donor and acceptor fluorophores and expressed in cells. Due to elevated expression levels, the p65 fusions were predominantly located in the nucleus for all constructs as can be inferred from figure 4 (panel b and e). The results of FLIM experiments performed using ECFP-p65 and EYFP-p65 are summarized in figure 4a. The average ECFP-p65 control phase and modulation lifetimes were 2.43±0.06 ns and 2.98±0.03 ns in agreement with ECFP lifetimes measured previously [10]. When EYFP-p65 was co-transfected, these values decreased only slightly to 2.32±0.08 ns and 2.86±0.05 ns respectively. Clearly, in these experiments it is very difficult to distinguish between a control and FRET situation.

Next, mKO-p65 and mCherry-p65 fusions were constructed and expressed in HeLa cells. Cells expressing mKO-p65 had a control phase and modulation lifetime of 3.56±0.13 and 3.76±0.03 respectively, similar to that observed for unfused mKO (fig. 4a,c,d). However, cells expressing both p65-mKO and p65-mCherry displayed a clearly decreased phase and modulation lifetime of 3.21±0.12 ns and 3.54±0.06 ns respectively which was homogeneously distributed over the nucleus (fig. 4a,f,g). The reduced donor lifetime demonstrates that the mKO and mCherry are in close proximity, indicating that the p65 subunit can homodimerize in living cells. Based on the phase lifetime, we detected 10% FRET for the mKO-mCherry pair and only 5% for the ECFP-EYFP pair.

## Discussion

In this study we systematically characterized yellow or orange fluorescent donors and red fluorescent acceptors for the detection of protein-protein interactions by FRET. These pairs offer several advantages, including excitation at higher wavelength, reducing autofluorescence and phototoxicity. Calculation of the R_0_ and expression of tandem constructs, demonstrated that the red-shifted pairs display a relatively high FRET efficiency, which can be detected by FLIM or acceptor photobleaching.

The characterization *in vivo* was performed by constructing tandem fusion proteins, since this gives control over donor to acceptor ratios (often not easy to achieve for two-component interaction studies). In addition, by choosing comparable linkers we tried as much as possible to obtain similar orientation and distance of the fluorophores in the tandem constructs. However, it should be kept in mind that the FRET efficiency in tandem constructs may not regularly depend on the length of the linker [33], since the addition or deletion of a few amino acids may change the orientation between donor and acceptor dipole moments, which is very important as discussed below.

While comparison between the constructs shown here can be made, the results cannot always be directly compared to FRET efficiencies obtained in other studies. First, the linkers we and others employ vary between constructs and consequently both distance and orientation can be different. To illustrate the effect of orientation on FRET efficiency within tandem constructs, circular permutated YFP variants were used as donors. There was a remarkable difference in FRET efficiency between constructs that have different circular permutated fluorescent proteins. Importantly, these results demonstrate that donor and acceptor fluorophores are not randomly oriented relative to each other, which is similar to what has been previously indicated [24]. Second, different methods are used across the literature to measure the FRET efficiency in tandem constructs of fluorescent proteins, giving rise to different apparent FRET efficiencies. For this reason we deliberately chose for the more robust and quantitative donor based methods, i.e. FLIM and acceptor photobleaching, to measure the FRET efficiencies in living cells. Acceptor based methods, e.g. so-called filter-FRET [34], [35], are complicated by direct excitation of the acceptor and bleed-through of donor fluorescence into the acceptor detection channel [25], requiring several correction factors. In addition, acceptor based FRET methods favor a high quantum yield of the acceptor to increase the signal of the sensitized emission, whereas for donor based methods, the acceptor quantum yield is irrelevant.

The relative high FRET efficiencies detected in single living cells are in agreement with large Förster radii calculated for red-shifted pairs (table 1), with values up to 64 Å for mKO-mCherry. This improvement can be explained by the relative high quantum yield of the yellow and orange donors and by the λ^4^ component in the overlap integral which generally increases the Förster radius for pairs in the red part of the visible spectrum. How do the red-shifted FRET pairs compare to other FRET pairs for detection of protein-protein interactions? The R_0_ values of the red-shifted pairs are higher than for green and yellow acceptors, including BFP-GFP and CFP-YFP (including the SCFP3A-SYFP2 FRET pair optimized in our laboratory, with an R0 of 54 Å [10]). Also others have tried to increase the FRET efficiency in the CFP-YFP pair. Using a FRET-based screen the FRET efficiency of a CFP-YFP construct was optimized, yielding the optimal CyPet-Ypet pair. However, this approach does not optimize the increase Förster radius since the extinction coefficients, spectra and quantum yields are not seriously changed [36]. The reason for the increase in the dynamic range of FRET-based caspase detection with the CyPet-YPet construct is the narrower spectrum of CyPet, decreasing donor bleed through and the probably more optimal orientation of the two fluorescent proteins in the tandem construct. Since nearly all of the point mutations found are on the surface of the fluorescent proteins, it is very likely that the optimal orientation is caused by a specific interaction between the CyPet and YPet beta barrels in one tandem construct [37]. Both the lack of intrinsically higher Förster radii and, more importantly, the possible interaction between CyPet and YPet proteins obstruct application of this pair for the study of protein-protein interactions. Especially for interaction studies it is essential to avoid any interaction between the fluorescent protein pair and an as high as possible Förster radius.

To increase the detection of FRET, YFP has been used as an acceptor for GFP fluorescence in FRET studies. Due to the high overlap, high quantum yield of the donor and high extinction coefficient of the acceptor the R_0_ is increased to 55 Å [38]. To reduce the complicated FRET analysis due to the strong overlap an elegant approach has been presented in which a non-fluorescent “dark” YFP-based acceptor was used (R_0_ = 59 Å) [39]. It was demonstrated that this pair was suited for detection of protein-protein interactions by FLIM, the downside being that expression of the acceptor cannot be quantified in living cells.

The first pairs that included red monomeric acceptors were based on EGFP as a donor, e.g. EGFP-mRFP1. Although EGFP-mCherry is an improved green red-pair (R_0_ = 54 Å) it still is significantly less efficient for FRET than the SYFP2-mCherry pair (R_0_ = 59 Å), due to a decreased overlap. Therefore, the use of yellow donors for FRET to mStrawberry and mCherry is preferred. Recently, a novel monomeric bright red fluorescent protein, tagRFP, was reported which is well suited as a FRET acceptor of EGFP, with an R_0_ of 58 Å [40]. Multimeric red acceptors can be used to raise the FRET efficiency (e.g. YFP/tandem-HcRed, R_0_ = 67 Å [41]) by increasing the extinction coefficient. Although this strategy should not be dismissed, e.g. one could also consider using tdTomato as an acceptor, tandem dimeric acceptors were not taken into account in this study, since we think it is preferable to only use monomeric fluorescent proteins, avoiding the increased size of the fusion protein.

The monomeric red-shifted FRET pairs described in this study show efficient FRET and have high R_0_ values. Therefore we think that SYFP2-mStrawberry, mKO-mCherry and mOrange-mCherry are the FRET pairs of choice for detecting protein-protein interactions by donor based quantitative FRET methods in living cells. The relatively low acceptor quantum yield will limit to some extent the use of the FRET couples described in this study for application in acceptor-based FRET applications such as FRET based biosensors, of which cameleon [42], [43] is the best-known example. For acceptor based FRET studies (e.g. ratio-imaging), the dynamic range of the response depends on the R_0_, the orientation of the donor and acceptor, direct excitation of the acceptor, bleed-through of donor fluorescence into the acceptor channel and the sensitivity of detection in the two channels that typically is variable in different setups. It is of note that, despite their optimal properties, the fluorescent proteins still need some attention. mStrawberry shows a relatively slow maturation, which can be solved by extending the period of the transient expression. mKO can be photoconverted, but only at high blue light intensities, which can be easily avoided.

The advantages of using a red-shifted pair are clearly demonstrated for the detection of homodimerization of the NF-κB subunit p65 by FRET. A twofold increase in FRET efficiency relative to the ECFP-EYFP pair was observed when the mKO-mCherry pair was used which reflects the increased R_0_ of this pair. In conclusion, we have shown that red-shifted FRET pairs are preferable for detecting protein-protein interactions by donor-based FRET methods in single living cells. The results of this study can serve as a guide for future FRET studies that are aimed at the detection of protein-protein interactions in living cells.

## Methods

### Construction of fluorescent protein fusions

mRFP1, mStrawberry, mCherry and mOrange were amplified from bacterial expression vectors (a kind gift of R.Y.Tsien) and used to replace the EGFP in the pEGFP-C1 vector (Clontech) for expression in mammalian cells. For mKO (kindly provided by A.Miyawaki) the same procedure was used, but in this case the last three amino acids of mKO (AHS) were replaced by the last three amino acids of GFP (LYK), which did not give fluorescent cells upon transfection. Therefore, two new constructs were made in which either the C- or both the N- and C-terminus were extended similar to what has been done for the fruit-series [16]. Both constructs gave orange fluorescent cells.

SYFP2 was amplified by PCR and inserted into pmRFP1-C1, pmStrawberry-C1 and pmCherry-C1 using KpnI and BamHI restriction sites. A similar strategy was used to construct pairs in which mOrange or mKO is the donor. Circular permutated Venus (cpV) variants (a kind gift of A.Miyawaki) were cut from YCam 3.20, YC3.30, YC3.60 YC3.70, YC 3.90 (abbreviated as cpV2, cpV3, cpV6, cpV7, cpV9 respectively) using SacI and EcoRI and inserted into pmCherry-C1 cut with the same enzymes. To obtain in-frame fusions, the resulting vectors were cut with BglII and subsequently 1 µg of cut vector was treated with 5 U mung bean nuclease (New England Biolabs) for 1 hr at 30°C to remove overhangs and ligated. All constructs were verified by sequencing. The plasmid encoding p65-EGFP was kindly provided of David Nelson [44]. EGFP was replaced by ECFP, EYFP, mCherry or mKO using AgeI and BsrGI restriction sites. For studies on dimerization of p65, cells were transfected with equal amounts of plasmid encoding p65-ECFP and p65-EYFP or p65-mKO and p65-mCherry. To obtain unquenched donor lifetime values, cells were single transfected with p65-ECFP or p65-mKO.

### Protein purification and fluorescence spectroscopy

His_6_-tagged proteins were produced in *E.coli* and purified on His-bind resin (Novagen, Darmstadt, Germany). After elution by imidazole the proteins were dialyzed 2x against PBS or 20 mM Tris. Spectral characterization was performed as described before with minor modifications [10]. For quantum yield determination, mOrange and mKO were diluted in 20 mM Tris (pH 8.0) 1 mM EDTA and compared to the standard Rhodamine 6G (Molecular Probes, Leiden, The Netherlands) in EtOH (QY = 0.94) [45], [46]. Excitation was at 515 nm (OD<0.05 nm) and emission was acquired over a wavelength range of 525–730 nm using a PTI Quantamaster 2000-4 spectrofluorometer (Photon Technologies International, Lawrenceville, NJ) and subsequently corrected for the instrumental response [47]. Samples were corrected for differences in absorbance at the excitation wavelength and for differences in refractive index (n = 1.329 for H_2_O and n = 1.359 for EtOH).

### Sample preparation

HeLa cells were transfected using 1 µl lipofectamine (Invitrogen, Breda, The Netherlands), 0.5 µg plasmid DNA and 50 µl OptiMEM per 35 mm dish holding a 24 mm Ø #1 coverslip. Unless specified otherwise, samples were imaged 2 days after transfection. Coverslips with cells were mounted in an Attofluor cell chamber (Invitrogen, Breda, The Netherlands) and submerged in microscopy medium (20 mM HEPES (pH = 7.4), 137 mM NaCl, 5.4 mM KCl, 1.8 mM CaCl_2_, 0.8 mM MgCl_2_ and 20 mM glucose). All measurements were done at room temperature.

### Fluorescence lifetime imaging microscopy

Fluorescence lifetime imaging was performed using the wide-field frequency domain approach on a home-build instrument [48] using a RF-modulated AOM and a RF-modulated image intensifier (Lambert Instruments II18MD) coupled to a CCD camera (Photometrics HQ) as detector. A 63x objective (Plan Apochromat NA 1.4 oil) was used for all measurements. The modulation frequency was set to 75.1 MHz. Twelve phase images with an exposure time of 50–100 ms seconds were acquired in a random recording order to minimize artifacts due to photobleaching [49]. An argon-ion laser was used for excitation at 514 nm, passed onto the sample by a 525 nm dichroic and emission light was filtered by a 545/30 nm emission filter. Each FLIM measurement is calibrated by a reference measurement of the reflected laser light using a modified filter cube [48] for correcting the phase and modulation drift of the excitation light. The reference is calibrated by averaging three to five FLIM measurements of a freshly prepared 1 mg/ml solution of erythrosine B (cat # 198269, Sigma-Aldrich, Zwijndrecht, The Netherlands) in H_2_O, which has a known short fluorescence lifetime of 0.086 ns [49], [50]. This extra calibration corrects for path-length differences and possible optics-related reflections that are different between the FLIM and reference measurements. At least five phase sequences were acquired from each sample. From the phase sequence an intensity (DC) image and the phase and modulation lifetime image are calculated [23] using Matlab macros. From these data, the average lifetime of individual cells is determined using ImageJ (http://rsb.info.nih.gov/ij/). Subsequently, average phase and modulation lifetimes (±standard deviation) are calculated. For the presentation of lifetime maps, a 5×5 smooth filter is applied to the raw data. The false-color lifetime maps and 1D and 2D histograms are generated by an ImageJ macro.

Photoconversion of mKO was performed by illuminating the sample for 10s with light from a 100 W Hg lamp filtered by a 436/20 nm filter. The power measured at the objective was 20 W/cm^2^.

The FRET efficiency E was calculated according to:
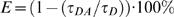
(3)in which τ_DA_ is the fluorescence lifetime of the donor in presence of the acceptor and τ_D_ is the fluorescence lifetime of the donor in absence of the acceptor. Since frequency domain FLIM yields a phase lifetime and a modulation lifetime, the FRET efficiency can be calculated based on τ_ϕ_ and τ_M_.

### Donor dequenching upon acceptor photobleaching

Donor dequenching upon acceptor bleaching studies were performed on a Zeiss LSM510 (Zeiss, Germany) confocal laser scanning microscope. A Zeiss 63x oil-immersion objective (Plan-Apochromat, NA 1.4) was used. YFP and RFP were excited using the 488 nm and 568 nm laser line respectively, which were reflected onto the sample by a 488/568 nm dichroic mirror. A secondary dichroic (570 nm) separated yellow and red fluorescence which were passed through a 505–550 nm bandpass and a 585 nm longpass filter respectively. A pinhole setting corresponding to 2 airy units was used and the multitrack (per frame) feature was used to ensure minimal crosstalk. Acquisition of images before and after bleaching was done with minimal laser excitation power (AOTF<0.5%) at zoom factor 2 whereas bleaching of red fluorescent protein was done at maximal power (100% 568 nm) with 150 iterations at zoom 2. The average SYFP2 fluorescence intensity of single cells before and after bleaching was quantified and the background was subtracted. These values were used to calculate the FRET efficiency E according to:
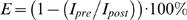
(4)in which I_pre_ is the average background-corrected fluorescence intensity of the donor before bleaching the acceptor, and I_post_ is the average background-corrected intensity of the donor after bleaching the acceptor.

## Supporting Information

Table S1 Amino acid sequences of the linkers that were used for the tandem constructs.
(0.04 MB DOC)
