# Supplementary material for: Sensitive Detection of p65 Homodimers Using Red-Shifted and Fluorescent Protein-Based FRET Couples
Source: PLoS One. 2007 Oct 10;2(10):e1011. doi: 10.1371/journal.pone.0001011 (PMC1995760; doi:10.1371/journal.pone.0001011)
Supplement: Table S1 — Amino acid sequences of the linkers that were used for the tandem constructs. (0.04 MB DOC) [file pone.0001011.s001.doc]

**Supplementary Information**

Goedhart *et al.* “Sensitive detection of p65 homodimers using red-shifted and fluorescent protein based FRET couples”.

**Table S1**. Amino acid sequences of the linkers that were used for the tandem constructs. The last 10 amino acids of the RFP C-terminus, the linker, and the first 10 amino acids at the N-terminus of the yellow or orange fluorescent protein are shown.

| **Tandem** | **RFP C-term** | **LINKER** | **YFP/OFP N-term** |
| --- | --- | --- | --- |
| mRFP-SYFP2 | RAEGRHSTGA | SGLRSRAQASNSAVDGT | VSKGEELFTG |
| mCherry-SYFP2 | STGGMDELYK | SGLRSRAQASNSAVDGT | VSKGEELFTG |
| mStrawberry-SYFP2 | STGGMDELYK | SGLRSRAQASNSAVDGT | VSKGEELFTG |
| mCherry-cpV2 | STGGMDELYK | SGLIEL | MTGKLPVPWP |
| mCherry-cpV3 | STGGMDELYK | SGLIEL | MQKNGIKANF |
| mCherry-cpV6 | STGGMDELYK | SGLIEL | MDGGVQLADH |
| mCherry-cpV7 | STGGMDELYK | SGLIEL | MLPDNHYLSY |
| mCherry-cpV9 | STGGMDELYK | SGLIEL | MITLGMDELY |
| mRFP-mOrange | RAEGRHSTGA | SGLRS | VSKGEENNMA |
| mCherry-mOrange | STGGMDELYK | SGLRS | VSKGEENNMA |
| mRFP-mKO | RAEGRHSTGA | SGLRSRAQASNSV | SVIKPEMKM |
| mCherry-mKO | STGGMDELYK | SGLRSRAQASNSV | SVIKPEMKM |
